# Commentary: Evaluation of post-COVID mortality risk in cases classified as severe acute respiratory syndrome in Brazil: a longitudinal study for medium and long term

**DOI:** 10.3389/fmed.2025.1558299

**Published:** 2025-05-16

**Authors:** Raphael Mendonça Guimarães, Christovam Barcellos, Renata Gracie, Diego Ricardo Xavier, Marcel de Moraes Pedroso, Monica de Avellar Figueiredo Mafra Magalhães

**Affiliations:** ^1^Department of Social Sciences, National School of Public Health, Oswaldo Cruz Foundation, Rio de Janeiro, Brazil; ^2^Health Information Lab, Science and Technology Information and Communication Institute, Oswaldo Cruz Foundation, Rio de Janeiro, Brazil

**Keywords:** COVID-19, COVID-19 vaccination, longitudinal analysis, mortality, severe acute respiratory syndrome, severe acute respiratory syndrome-related coronavirus 2 (SARS-CoV-2)

## Introduction

We have read with concern the article titled “Evaluation of post-COVID mortality risk in cases classified as severe acute respiratory syndrome in Brazil” (1). The authors conducted a retrospective cohort study using data from Brazil's Epidemiological Surveillance System (SIVEP), covering 2020 to 2023. They analyzed cases of Severe Acute Respiratory Syndrome (SARS) attributed to COVID-19, including demographic information, comorbidities, vaccination status, number of doses received, municipality of residence, and survival outcomes. To assess mortality risks in the medium and long term after infection, the researchers applied classical Cox, mixed-effects, and frailty models. The analysis considered two periods: medium-term (3 months to 1 year after symptom onset) and long-term (>1 year after symptom onset). The results indicated that, in the medium term, vaccination reduced the risk of death by 8%, whereas in the long term, the risk of death nearly doubled for vaccinated individuals. Turns out, the article's main message suggests that the protective effect of COVID-19 vaccination was reversed after 1 year, indicating an increased risk of death for vaccinated individuals. In response, we would like to offer some methodological contributions and provide a more contextualized reflection on the implications of these results.

First, it is important to recall that Brazil was one of the epicenters of the COVID-19 pandemic (2). The pandemic response in Brazil was surrounded by controversies, including the federal government's denial of the pandemic's severity (3, 4), inefficiencies in organizing healthcare services—such as inadequate contact tracing for asymptomatic individuals and limited availability of intensive care unit beds for severe cases (5)—and delays in initiating vaccination efforts (6, 7). This context is crucial to understanding the negative impact of the weak evidence presented by Rodrigues and Andrade (1), particularly given the potential for their findings to spread misleading information. Regarding the methodology employed by the authors, we would like to address several points.

## Discussion

Concerning the data source, we emphasize that the SIVEP-Gripe system, designed for epidemiological surveillance of severe respiratory syndromes, is insufficient for a robust analysis of mortality. A more appropriate analysis would require examination of general mortality data within the population, which could be achieved using the Mortality Information System (SIM). Utilizing SIM data alongside SIVEP-Gripe would undoubtedly enhance accuracy in identifying causes of death and reduce classification bias (8).

With respect to the study's variables, the data source is unique for collecting all variables. Due to that, critical elements for rigorous analysis were omitted. Factors such as the type of vaccine administered, the timing of vaccination relative to hospitalization, delays in vaccination, and the vaccination regimen employed are essential for precise classification of participants' vaccination status and, indirectly, their serological condition. Therefore, the precariousness of the information, for example, on vaccination status, which is the study's primary outcome, is notorious. The fact that this information is implicitly self-reported introduces information bias. Once again, we highlight that SIVEP-Gripe is not the most suitable information system for this purpose; rather, the National Immunization Program Information System (SI-PNI) should be utilized.

Regarding the statistical model, while the authors employed adjusted Cox models for longitudinal analysis, there was inadequate adjustment for key confounders, particularly concerning healthcare access and infrastructure at subnational levels. The Brazilian federal system, along with its healthcare policies, operates through three levels—municipal, state, and federal—and includes regional organization (9). This system exhibits remarkable structural diversity, ranging from municipalities with only basic healthcare units to others with populations and infrastructure exceeding those of entire states (10). Such diversity has significant implications for health outcomes. Finally, an evaluative study should use additional techniques to simulate scenarios and study latent variables. The Cox model is not sufficient for this. Instead, a microsimulation study, or the use of propensity score could be very useful to fulfill this lac.

Moreover, as a hospital-based longitudinal study, there is a possibility of survival bias, given that many individuals died before being admitted to a hospital, especially during critical phases of the pandemic when Brazil's healthcare system collapsed due to shortages of ICU beds, mechanical ventilation equipment, and even oxygen support for severe respiratory cases (11). Still, regarding the study population, we consider it essential to emphasize that there is potential selection bias, considering the eligibility criteria for the deaths analyzed. The exclusion criteria remained unclear in the text, which does not allow for a more qualified analysis of this type of bias.

Additionally, non-vaccinated individuals who survive severe acute respiratory syndrome (SARS) may have better clinical conditions, while vaccinated individuals who develop SARS might represent a more vulnerable population. The exclusion of non-hospitalized cases further limits the understanding of the full impact of vaccination. Furthermore, the study failed to adequately address potential comorbidities and socioeconomic and structural inequalities, despite evidence that these factors were significant determinants of the pandemic's severity in Brazil (12).

For these reasons, a longitudinal analysis that does not account for such critical exposure characteristics or specific causes of death provides a statistical correlation that does not necessarily imply causation. It is worth mentioning that considering the nature of the outcome (vaccine efficacy), alternative study designs could have been not only more efficient but also more representative, reducing selection bias. Case-control or case-cohort designs, for instance, might have been better suited for this investigation (13).

What truly occurred was that the management of the COVID-19 pandemic in Brazil, fueled political tensions, negatively impacted the healthcare system, and triggered social despair. Part of this scenario was due to the spread of fake news and the constant questioning of vaccine efficacy. It resulted in nearly 1 million deaths while COVID-19 was classified as a public health emergency of national interest (14). Therefore, vaccination should not be described as a measure that causes higher mortality in the medium and long term.

On the contrary, the pandemic data supports vaccination. In the years 2020, 2021, 2022, and the first half of 2023, the COVID-19 mortality rates per 100,000 inhabitants were 10.26, 16.45, and 0.14, respectively. In the same chronological order, the hospitalization rates for COVID-19 were 28.96, 47.04, and 0.40 per 100,000 inhabitants. It is noteworthy that both hospitalizations and deaths drastically decreased when the first-dose vaccine coverage reached 90% (15). The pandemic highlighted the need for coordination between different levels of government and the importance of clear communication with the population to contain the spread of the virus. Preparedness and response to future public health emergencies depend on the population's understanding of the benefits of vaccination. Furthermore, it is essential to emphasize how denialism was central to explaining the deaths of tens of thousands of people (16). For this reason, we present this counterpoint to the results published by Rodrigues and Andrade (1).

In summary, the causal relationship suggested by the authors is inadequate due to the limitations outlined. The dataset used lacks representativeness of the general population, compromising the study's external validity. On the other hand, the absence of critical information needed for proper classification of the “exposed” and “unexposed” groups undermines its internal validity. These combined limitations render the results purely speculative, which could lead to misinterpretations and recommendations against COVID-19 vaccination without robust evidence.
